# Rainfall changes affect the algae dominance in tank bromeliad ecosystems

**DOI:** 10.1371/journal.pone.0175436

**Published:** 2017-04-19

**Authors:** Aliny Patricia Flauzino Pires, Juliana da Silva Leal, Edwin T. H. M. Peeters

**Affiliations:** 1 Departamento de Ecologia, Instituto de Biologia, Universidade Federal do Rio de Janeiro, Rio de Janeiro, Brazil; 2 Fundação Brasileira para o Desenvolvimento Sustentável, Rio de Janeiro, Brazil; 3 Brazilian Research Network on Climate Change—Rede Clima, Instituto Nacional de Pesquisas Espaciais, São José dos Campos, São Paulo, Brazil; 4 Department of Environmental Sciences, Aquatic Ecology and Water Quality Management Group, Wageningen University, Wageningen, The Netherlands; University of Minnesota, UNITED STATES

## Abstract

Climate change and biodiversity loss have been reported as major disturbances in the biosphere which can trigger changes in the structure and functioning of natural ecosystems. Nonetheless, empirical studies demonstrating how both factors interact to affect shifts in aquatic ecosystems are still unexplored. Here, we experimentally test how changes in rainfall distribution and litter diversity affect the occurrence of the algae-dominated condition in tank bromeliad ecosystems. Tank bromeliads are miniature aquatic ecosystems shaped by the rainwater and allochthonous detritus accumulated in the bases of their leaves. Here, we demonstrated that changes in the rainfall distribution were able to reduce the chlorophyll-*a* concentration in the water of bromeliad tanks affecting significantly the occurrence of algae-dominated conditions. On the other hand, litter diversity did not affect the algae dominance irrespective to the rainfall scenario. We suggest that rainfall changes may compromise important self-reinforcing mechanisms responsible for maintaining high levels of algae on tank bromeliads ecosystems. We summarized these results into a theoretical model which suggests that tank bromeliads may show two different regimes, determined by the bromeliad ability in taking up nutrients from the water and by the total amount of light entering the tank. We concluded that predicted climate changes might promote regime shifts in tropical aquatic ecosystems by shaping their structure and the relative importance of other regulating factors.

## Introduction

Ecosystems face many concurrent stressors which may cause great changes in their functioning. It has been proposed that climate change and biodiversity loss could promote large shifts in natural ecosystems compromising important ecological services [1–3]. Also, tropical aquatic ecosystems are affected by these two stressors since most of them are only fed by rain and because these ecosystems are prone to high rates of biodiversity loss [4].

Climate models predict that changes in rainfall distribution will be the prevalent change across different areas of the globe, but especially in tropical and high latitude areas [5]. Extreme rainfall events may cause unpredicted changes in aquatic ecosystems and may ease a return to a pristine condition in natural environments [6,7]. Changes in rainfall distribution, such as longer periods of drought followed by extreme rainfall events, may lead to a decrease in water level in lakes over a longer period of time. This might favor the development of submerged aquatic plants and reduce algal growth leading to a shift towards a pristine state [8]. Controlling local stressors has been proposed as one of the most effective ways to prevent ecosystems to shift from one condition to another due to climate changes [1,9].

Biodiversity loss is considered one of the major drivers of the current global changes [2]. On the other hand, biologically diverse systems may ensure ecosystems against disturbances and can make them more resistant to the effects of climate changes [10–13]. Species-rich ecosystems seem to be more stable to environmental changes than species-poor systems through various mechanisms, including asynchrony and compensatory population dynamics, different resistance strategies and portfolio effects [14,15]. In this way, biodiversity may favor the maintenance of a pristine condition, especially given the predicted climate change [10]. In aquatic ecosystems, leaf litter is an important food source and substrate and its diversity can provide insurance face to climate changes [16–18]. Empirical approaches could help to identify the underlying mechanisms and to reveal potential interactions between biodiversity and climate change [15,19]. However, experiments unraveling how climate changes and biodiversity jointly drive natural aquatic environments are scarce probably due to the great challenge in manipulating whole ecosystems.

Alternatively, tank bromeliads have been increasingly used as model ecosystems to test many ecological questions [16,20–23]. These natural microcosms are miniature aquatic ecosystems that are shaped by rainwater and allochthonous detritus accumulated in the bases of their leaves. They have great experimental potential due to their handling and replication ability [20]. Empirical studies indicate that these ecosystems can be dominated by two different food sources. Primarily, tank bromeliads ecosystems would be sustained by the allochthonous detritus from the surrounding vegetation (i.e.:“brown” condition); or alternatively, by the autochthonous organic matter, mainly algae growing in the water tank (i.e.:“green” condition) [24,25]. Multiple environmental factors are associated with this green and brown conditions and the importance of algae in these systems has been more and more recognized in the last years [25–28]. Studies suggest that bromeliad size, the amount of organic matter and light inputs are the most important factors to determine algae biomass in these ecosystems [24,26,29]. Increases in bromeliad size can ensure the maintenance of an aquatic condition favoring algae colonization and allowing higher decomposition rates [26] which may be beneficial for the development of an algae-dominated condition. Occasional inputs of organic matter will also regulate algae biomass in tank bromeliads by controlling nutrients concentrations therein [29]. Recently, it has also been demonstrated that light input can regulate algal biomass in tank-bromeliad ecosystems and explain the relative importance of the allochthonous contribution to the system [24]. Ecosystems with greater light inputs had their food webs more closely related to algae than those less exposed to sunlight [24].

As these aquatic ecosystems are fully fed by rainwater, changes in rainfall could disrupt important mechanisms that regulate algae growth therein. For example, increases in rainfall can raise the water level in tank bromeliad ecosystems [30] and it can favor the light entrance in the tank or speed up litter decomposition which benefits algae dominance. Controversy, rainfall itself can dilute or even overflow the water in the bromeliad tanks which may decrease nutrients concentration and algae in the water. Additionally, bromeliad plants are also able to take up nutrients from the water in their tanks [31,32]. Thus, the effects of rainfall changes on the occurrence of a green condition may be mediated by the ability of tank bromeliads in removing nutrients from the water. However, the interaction between algae and bromeliads and how they regulate the functioning of phytotelma are considered open questions in the ecology of these ecosystems [33]. Empirical studies using isotopic analysis could solve the main gaps in the relationship between bromeliad nutrition and the water in their tank, including the potential competition by nutrients with algae [33].

In this study, we experimentally test the effects of rainfall changes and biodiversity on the occurrence of algae-dominated conditions in tank bromeliads ecosystems. We controlled biodiversity by manipulating litter diversity in the bromeliads tanks and using all combination of three different species from our field site. Then, we manipulated temporal clustering and amplitude of rainfall by using five different rainfall scenarios predicted to occur in the Southeastern Brazil. We used stable isotopes analysis to verify the potential effect of rainfall changes in regulating the ability of bromeliads in uptaking nutrients from the water in their tank. We hypothesized that (i) changes in rainfall distribution will decrease the occurrence of an algae-dominated condition by suppressing the ability of bromeliads in taking up nutrients from their tanks and (ii) this effect will be minimized by the positive effects of litter diversity on the algae dominance. At last, we discuss whether the occurrence of an algae-dominated condition in tank bromeliads can be regarded as alternative states by providing a conceptual framework based on the actual knowledge about these ecosystems.

## Material and methods

### Experimental design

**Fig 1:**
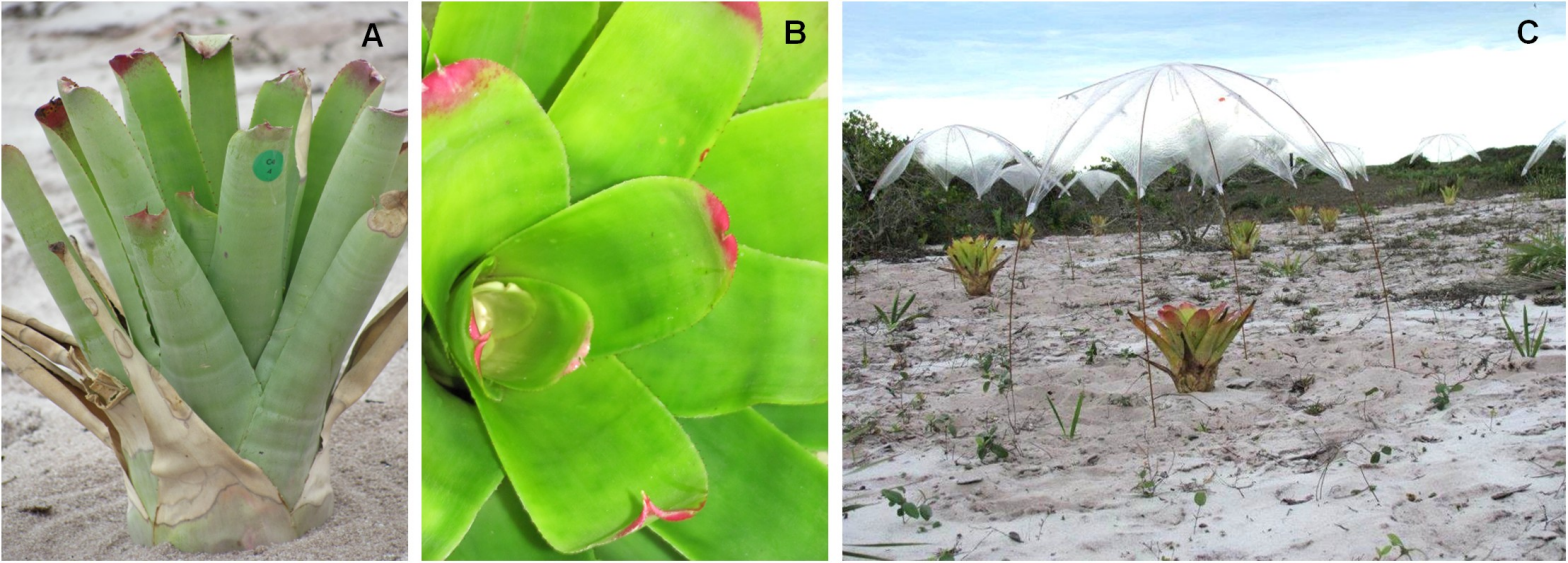
The tank bromeliad *Neoregelia cruenta* used in the current experiment. (A) Detail of the central tank that hosts the miniature aquatic ecosystem created by the rain water accumulated between the bromeliad leaves (B). Panoramic view of the experiment after shelter installation (C). Each bromeliad was in the field for 84 days and received water as described by their rainfall scenario treatment (300 mm). Experimental details are described in Material and Methods.

We experimentally manipulated the amount of water and litter diversity input for the tank bromeliad *Neoregelia cruenta*(Graham) L. B. Sm. (Fig 1), in the Restinga de Jurubatiba National Park, Rio de Janeiro State, Brazil (- 22°14’18”S, 41°33’35”W). The Restinga de Jurubatiba National Park is characterized by shrubby vegetation patches in a sandy soil matrix. We used a full-factorial design, composed of five rainfall scenarios crossed with all combinations of three litter species (seven litter combinations, including all 1-, 2- and 3-species combinations), resulting in 35 treatments with five replicates each (175 experimental units). All samplings and procedures were conducted according to the national and international guidelines and licensed by the Instituto Chico Mendes de Conservação da Biodiversidade, Ministério do Meio Ambiente (research permit 26995-1/2011).

We established the rainfall scenarios based on precipitation projections for Southeastern Brazil for the second half of this century (2071 to 2100). These projections include an increase in temporal clustering of rainfall and in the occurrence of extreme rainfall events, but no changes in the total amount of rain [34]. We established an *Ambient* rainfall scenario based on the daily rainfall pattern over the rainy season (November to February) for our field site during nine years (1997 to 2005; Instituto Brasileiro de Meteorologia, INMET). *Ambient* rainfall scenario was characterized by a 25 mm rainfall event with a six days interval between rainfall events, distributed over 84 days (12 rainfall events) with a total 300 mm of rainfall. For the altered rainfall scenarios, we held the total amount of rainfall added constant (300 mm) but increased the temporal clustering of rainy days (two “clustering” scenarios) and the occurrence of extreme rainfall events (two “amplitude” scenarios). In the clustering scenarios, we decreased the interval between rainfall events; clustering rainfall either as two consecutive rainy days (25 mm per rainy day; 50 mm per two-day event) with a 12-day interval between rain events in a *Medium Clustering* scenario (MC scenario), or as four consecutive rainy days (25mm per rainy day; 100 mm per four-day event) with a 24-day interval between rain events in a *High Clustering* scenario (HC scenario). For the amplitude scenarios, we increased the intensity of each rainfall event and the interval between events. In a *Medium Amplitude* scenario (MA scenario), a one-day 50 mm rainfall event occurred every 14 days, while in a *High Amplitude* scenario (HA scenario), a one-day 100 mm rainfall event occurred every 28 days. As we intentionally constrained our rainfall manipulations to have total equivalent amounts of rain, each altered rainfall scenario necessarily altered several rainfall variables simultaneously. For example, increases in the occurrence of extreme events (*MA* and *HA* rainfall scenarios) resulted in more consecutive days without rain, as it is predicted to occur [35]. For more details about rainfall manipulation, including rainfall scenarios and the watering procedures related to our experimental design, see [16].

We manipulated litter diversity by choosing plants from different metabolic groups (C_3_ –*Eugenia uniflora*, C_4_ –*Cyperus sp*. and CAM–*Clusia hilariana*) that are potential litter source for tank bromeliads at our field site. We collected C_4_ and CAM litter in the field and then washed and dried at 60°C until its mass stabilized (at least 48 hours). *E*. *uniflora* was cultivated in a greenhouse with controlled irrigation for ^15^N enrichment (see next paragraphs). Each bromeliad received 1.2 g dry weighted of its respective litter combination. We choose this amount of litter because it is comparable to the natural amounts found in the main tank of *N*. *cruenta* bromeliads (unpublished data). We accommodate the untied litter in the bromeliad central tanks because they are more susceptible to the other factors that regulate algal biomass in the tank bromeliads (i.e.: light input, hydrology). In total, all 1-, 2- and 3-species combinations (seven litter combinations) were applied through a substitutive design. It means that all experimental units received the same amount of litter but in different proportions of species. The 1-specie treatments (C_3_, C_4_ and CAM) received 1.2 g of litter from the same litter species; the 2-species treatments (C_3_-C_4_, C_3_-CAM and C_4_-CAM) received 0.6 g of each litter species in the treatment; and in the 3-species treatment (C_3_-C_4_-CAM), we added 0.4 g of each litter species.

All bromeliads were dug up and washed to remove all organisms to standardize initial conditions. As bromeliad size is a strong determinant of bromeliad water dynamics [36], we controlled bromeliad size effects in several ways: (i) by choosing bromeliads of approximately 30 cm in diameter; (ii) by ensuring that any residual variation in size was evenly distributed between treatments, and (iii) by measuring the maximum volume of each bromeliad and using this as a covariate in statistical analyses. We measured the maximum volume of each tank bromeliad by slowly filling them with water using a volumetric becker until they overflowed [26].

Bromeliads were replanted in the “*restinga*” in a spatially randomized arrangement three months prior to the rainfall manipulation. Each bromeliad received its respective litter treatment and was weekly water-filled during these three months (September to November 2011) to allow community assembly. After this period, each bromeliad was covered with an individual transparent shelter to prevent natural inputs of rain but which allowed light to enter the bromeliads tank as well as organisms’ colonization in these systems (Fig 1C). The rainfall scenarios were simulated experimentally for 3 months during the rainy season (November 2011 to February 2012) by adding mineral water to each bromeliad using a watering can, according to its specific rainfall scenario. We determined the total volume of water to be added in each bromeliad based on its mean surface catchment area [16]. All response variables were sampled at the end of the experiment, five days after the last rainfall event. We sampled at this moment because it allows the system to recover its own dynamic after rainfall disturbance but it still retains water for sampling. Additionally, we decided to sample at the end of the rainfall manipulation to avoid resetting important ecological processes.

We verified the effects of rainfall changes and litter diversity on algae biomass by measuring the chlorophyll-*a* content in the water of the bromeliads tank. We collected the water by turning the bromeliad upside down in a bucket which allowed us to homogenize the water from all tanks, as previously suggested [37]. Then, we filtered 40 ml of water in 0.7 μm pore size cellulose filters that were kept frozen until analysis. Chlorophyll-*a* concentration was determined by spectrophotometry at 665 nm, after extraction with ethanol 90% at 80°C [38]. Recently, it has been reported an unexpected concentration of bacteriochlorophyll-*a* in tank bromeliads ecosystems [39]. Thus, it is possible that part of our measurements does not report only algal but also bacteria biomass. However, as we used 0.7 μm pore size cellulose filters, we consider that most of the bacteriochlorophyll-*a* was not retained in the filter. Also, the relationship between algal biomass and chlorophyll-*a* is widespread and consistent in the ecological literature and chlorophyll-*a* is still considered one of the best proxy measurements of algal biomass in aquatic ecosystems, including tank bromeliads [26,28,40].

Studies in temperate shallow lakes usually assume that algae-dominated systems have chlorophyll-*a* concentration higher than 20 μg L^-1^[41–43] whereas in tropical shallow lakes this is 40 μg L^-1^[44]. However, the total amount of water in tank bromeliads may vary greatly which could lead to a misread in the identification of an algae-dominated condition. Thus, we used the distribution of chlorophyll-*a* concentration in the *Ambient* rainfall scenario (S2 Fig) to establish which value identify a different group of ecosystems. We concluded that 80 μg L^-1^ is a reasonable and conservative value to determine the occurrence of an algae-dominated condition. *In situ* visual observations were regularly done during the experiment to verify the temporal consistency of the rainfall scenario and litter diversity effects on determining the occurrence of algae-dominated ecosystems. For methods, data analysis and main results of the qualitative approach see Supporting Information.

We measured nutrients, color and turbidity to verify whether the occurrence of an algae-dominated condition was regulated to changes in these environmental variables. Nutrient concentration and water color were assessed using the filtered water in 0.7 μm pore size cellulose filters (Advantec). Water color was determined spectrophotometrically by light absorption at 430 nm in 1 cm cuvette [45]. The concentrations of dissolved nitrogen and carbon in the water were estimated by using a carbon analyzer (TOC-V CPN, Shimadzu) with a nitrogen analyzer module coupled (TNM-1, Shimadzu). The phosphorus concentration was obtained by spectrophotometry using the colorimetric method with molybdic acid [46]. Turbidity was assessed by measuring the intensity of light scattered using an HI 98703 Hanna turbidimeter.

We used ^15^N labeled leaves of *E*. *uniflora* in all treatments containing C_3_ litter to determine the indirect effects of rainfall on the ability of bromeliad leaves in removing nutrients from their tank water. However, we only analysed these effects in the C_3_- litter treatments because i) we were mainly interested in the effects of rainfall in regulating the ability of bromeliads in determining the nutrient loading of their tank and ii) we only have ^15^N enriched *E*. *uniflora*, therefore in the mixed treatments several biases could occur associated with nitrogen competition during bromeliad acquisition of nutrients by unbalanced ^14^N/^15^N ratios among litter species. Thus, our experimental design did not allow us to verify the potential effects of litter diversity on the bromeliad ability to uptake nutrients from their tank because this manipulation is restricted to C_3_ litter treatments.

### Data analysis

We verified the occurrence of different conditions in tank bromeliads by using the bimodality approach [44]. This method is recommended and very useful in studies with limited temporal data [44], such as the present experiment. We tested which type of distribution fitted best to the chlorophyll-*a* data following the recommendations and procedures are given in literature [44]. We used the chlorophyll-*a* concentration values found in the last sampling to perform this analysis. Bimodality was demonstrated using the Bayesian Information Criterion (BIC) values [44]. The lowest BIC value indicates the best distribution for the number of classes used for each variable. For the full description of the bimodality approach, see [44].

We analyzed the effects of bromeliad maximum volume, rainfall scenario, litter diversity and their interaction on the occurrence of algae dominance by using Generalized Linear Models (GLMs). We also used GLMs to evaluate the effects of water color, turbidity, dissolved nitrogen, dissolved phosphorus and dissolved organic carbon on the occurrence of an algae-dominated condition. For these analyses, we used a binomial distribution and designated the algae-dominated bromeliads by the value “1” and non algae-dominated bromeliads by the value “0”. To check the correlation between chlorophyll-*a* and the ^15^N content in the bromeliad leaves, we performed linear models to test the effect of rainfall scenario on the ^15^N isotope ratio content in the bromeliad leaves.

All models were performed using R v. 3.2.2 [47]. We carried out the LMs with base functions in R. We obtained the BIC values for the chlorophyll-*a* data in each rainfall scenario using the “*flexmix”* package.

## Results

**Fig 2:**
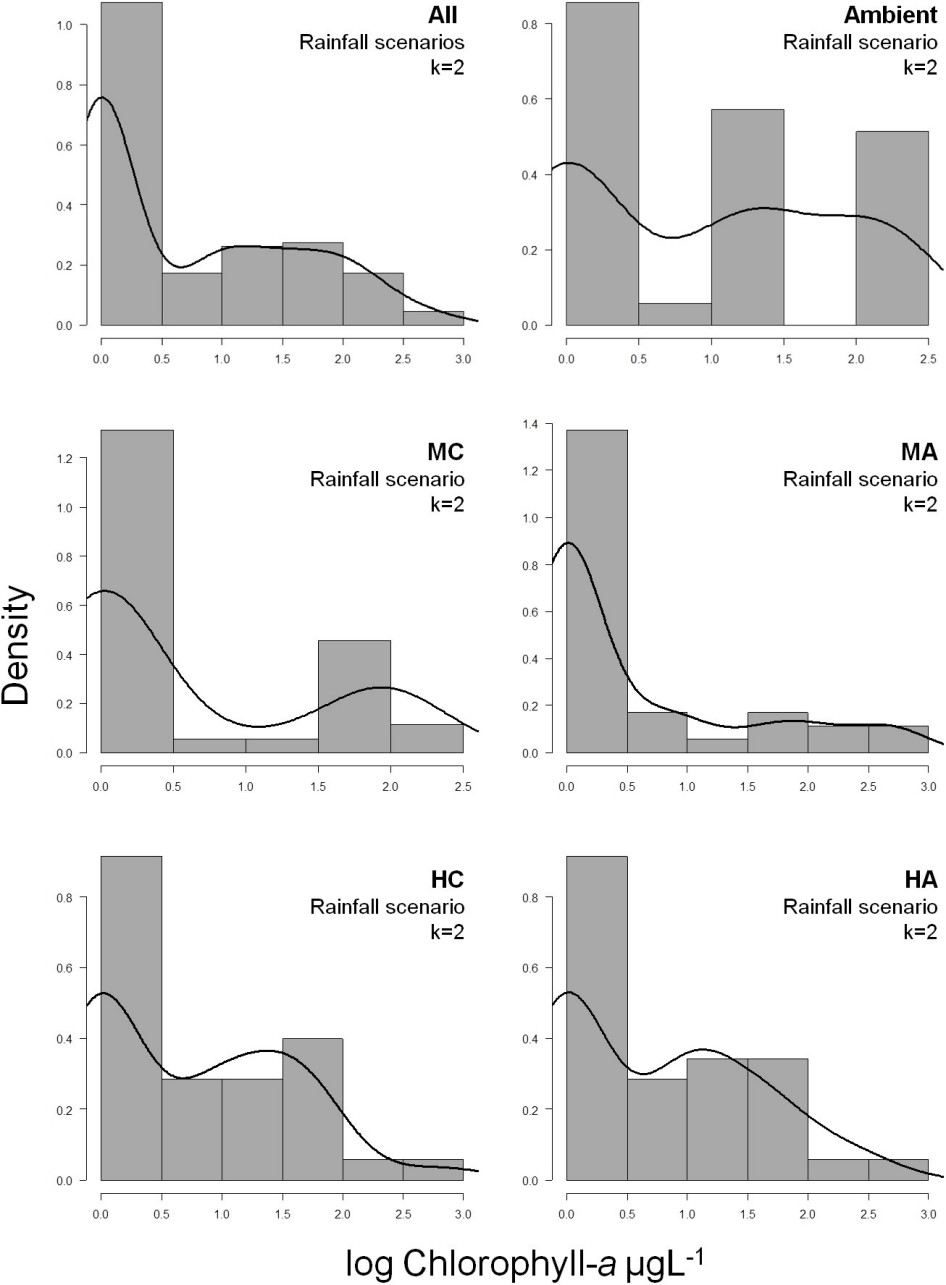
Frequency distribution of log-transformed chlorophyll-*a* (μg L^-1^) in the water of tank bromeliads. Frequency distribution was determined for all rainfall scenarios (n = 175) and *Ambient*; Medium clustering (*MC*), Medium amplitude (*MA*), High clustering (*HC*) and High amplitude (*HA*) rainfall scenarios separately (n = 35). The lines depict the probability density described by the best fitting model resulting from the BIC analysis, represented by the k values in the panels. For statistical details see Table A in S1 Appendix.

In all rainfall scenarios and litter diversity treatments, the non algae-dominated condition occurred in at least 70% of all tank bromeliads (Fig 2). Furthermore, the BIC values used in the bimodality approach showed that in all rainfall scenarios two classes best described the distribution of chlorophyll-*a* (Fig 2). We were able to identify one class of bromeliads with very low chlorophyll-*a* concentration and another class with values higher than 20 μg L^-1^ (Fig 2, S1 Fig). The *Ambient* rainfall scenario showed a representative group algae-dominated bromeliads (chlorophyll-*a* higher than 80 μg L^-1^; Table A in S1 Appendix; Fig 2), but changes in rainfall distribution decreased the occurrence of them in the HA and HC rainfall scenarios (Table 1; Fig 2).

**Table 1:** Rainfall effects on algae-dominated conditions. General linear model for the effects of bromeliad volume, rainfall scenario, litter diversity and the interaction between rainfall and litter diversity on the occurrence of algae-dominated conditions. Algae-dominated ecosystems were defined as those which water contained more than 80 μg chlorophyll-*a* L^-1^. Rainfall scenarios *Ambient*, Medium clustering (*MC*), Medium amplitude (*MA*), High clustering (*HC*) and High amplitude (*HA*) are described in the main text. Significant values are presented in bold numbers (*P*<0.05).

Factors	d.f	χ^2^	*P*
Bromeliad volume	1	0.002	0.963
Rainfall scenario (RS)	4	**9.75**	**0.045**
Litter diversity (LD)	1	1.20	0.273
RS x LD	4	6.14	0.189

**Fig 3:**
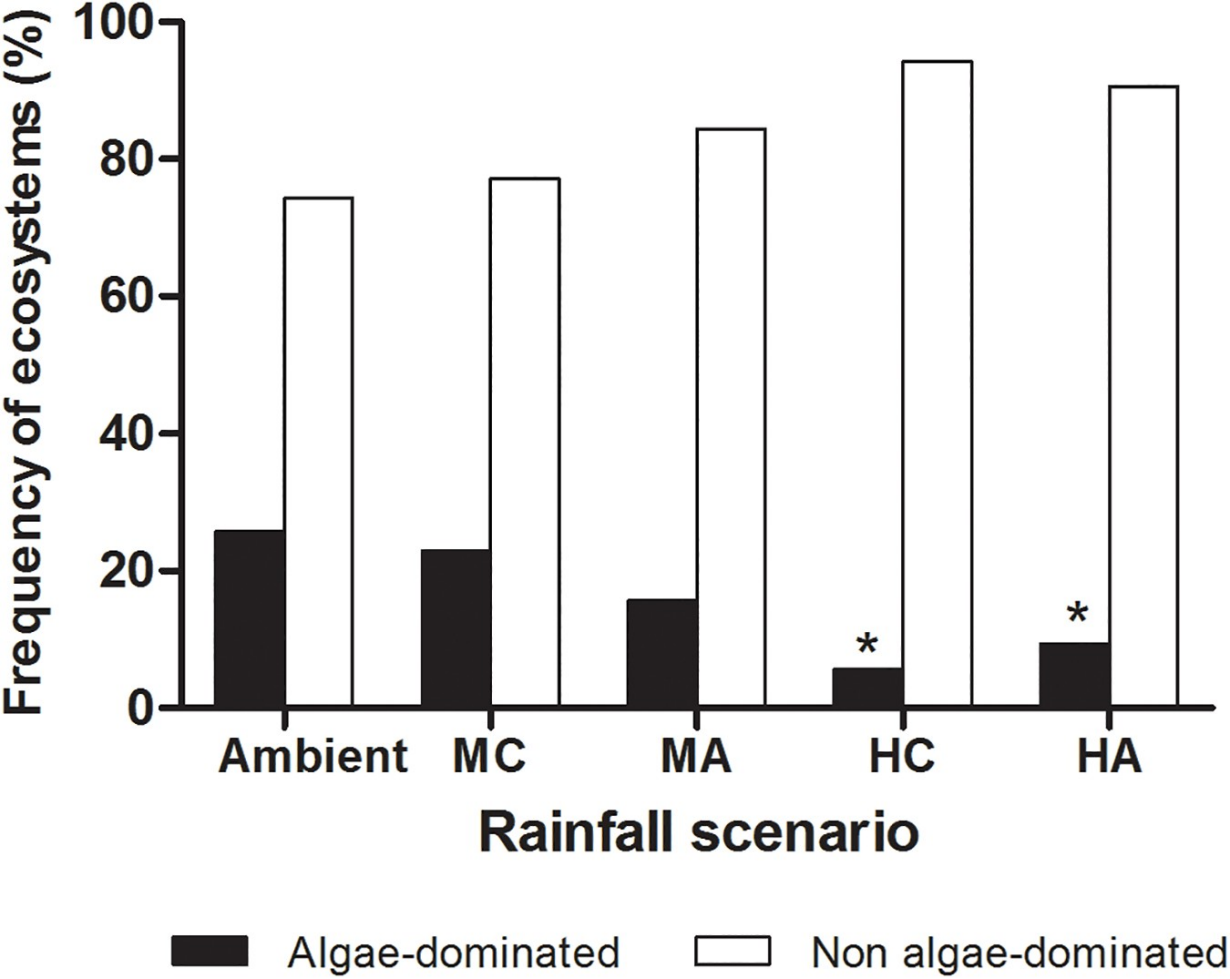
Effects of rainfall scenarios on the frequency of algae-dominated tank bromeliads ecosystems. Algae-dominated ecosystems were characterized as those contained more than 80 μg L^-1^. An asterisk (*) means significant differences from *Ambient* rainfall scenario (*P*< 0.05). Rainfall scenarios–*Ambient*, Medium clustering (*MC*), Medium amplitude (*MA*), High clustering (*HC*) and High amplitude (*HA*) rainfall scenarios, n = 35. For statistical details see Table 1.

Rainfall changes affected the algae dominance in tank bromeliads (Table 1, Fig 3) with a significantly lower number algae-dominated conditions in *HA* (z = -1.993, *P* = 0.046) and *HC* rainfall scenarios (z = -2.11, *P* = 0.034). In *Ambient* rainfall scenario, the number of bromeliads dominated by algae was up to 5 folds greater than in *HC* rainfall scenario (Fig 3). Litter diversity, its interaction with rainfall scenario and bromeliad size did not affect the number of algae-dominated ecosystems (Table 1). Additionally, we did not observe any significant effect of water color, turbidity, dissolved nitrogen, dissolved phosphorus and dissolved organic carbon on the occurrence of algae-dominated conditions (Table B in S1 Appendix; For all factors *P*>0.36).

**Fig 4:**
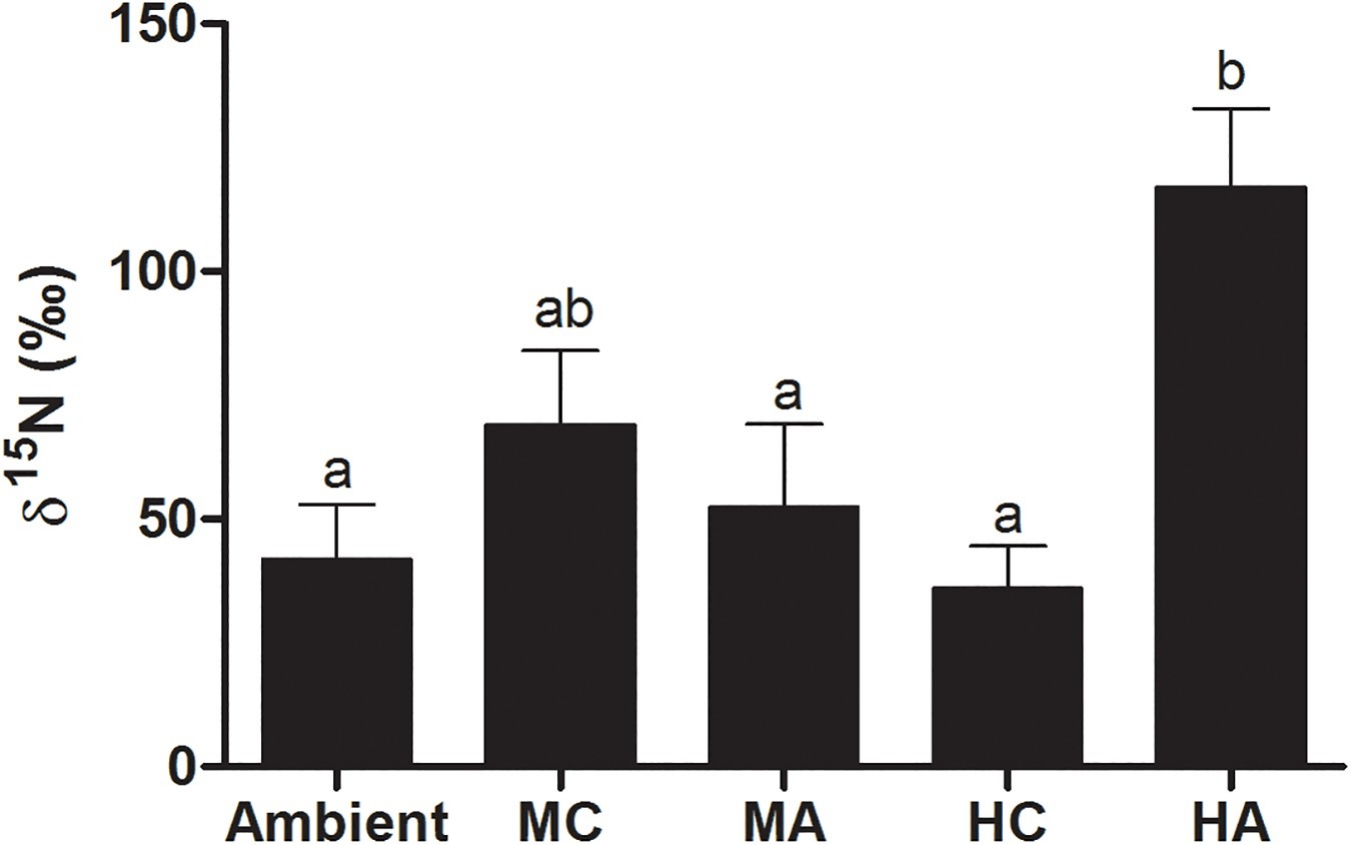
δ^15^N of *Neoregelia cruenta* leaves after receiving enriched leaves of *Eugenia uniflora* under the following distinct rainfall scenarios: *Ambient*, Medium clustering (*MC*), Medium amplitude (*MA*), High clustering (*HC*) and High amplitude (*HA*). Bars indicate standard errors of means. Different small letters indicate statistical differences among treatments (*P*< 0.05).

We found a significant effect of rainfall scenario on the δ^15^N content in bromeliad leaves (F_4,44_ = 5.33, *P* = 0.0013; Fig 4). Tank bromeliads from *HA* rainfall scenario had almost three times higher amount of δ^15^N in their leaves than observed in *Ambient* rainfall scenario (Fig 4). In C_3_ litter treatments, we also observed a negative correlation between the δ^15^N in the bromeliad leaves and chlorophyll-*a* concentration in the water sampled at the end of the experiment (F_1,46_ = 4.66, *P* = 0.036; best-fit slope = -0.7686 ± 0.3562).

## Discussion

The understanding of how ecosystems shift to critical conditions is a challenging question for ecosystem conservation and management in the face of global changes [1,7]. Our results demonstrated that rainfall changes decreased the occurrence of algae-dominated ecosystems, mainly at *HA* and *HC* rainfall scenario, but litter diversity was not able to mediate this effect. Here, we suggest that predicted changes in rainfall will affect algal biomass in tank bromeliad ecosystems by regulating the ability of bromeliad in uptaking nutrients from the water in their tanks.

We demonstrated that the non algae-dominated condition occurred most in the tank bromeliads *Neoregelia cruenta*. In all rainfall scenarios, we observed a large group of bromeliads where chlorophyll-*a* concentration was below detection level and a smaller group with a detectable source of algae in the water of their tanks. Particularly, in *Ambient* rainfall scenario, we observed a bimodal distribution of chlorophyll-*a* with many observations around zero and a number of observations above 80 μg L^-1^. Previous studies using the same tank bromeliad, but at natural conditions and containing different amounts of litter, found a much lower maximum chlorophyll-*a* concentration, around to 27 μg L^-1^ [26,48]. We suggest that the difference with previous studies is related to the light input in the tanks since light availability seems to be an important determinant of primary productivity of these ecosystems [24]. Our experiment was performed in an open area without surrounding vegetation and canopy cover and thus ensuring that each experimental unit received the same and maximum light input (no dossel cover, Fig 1C).

**Fig 5:**
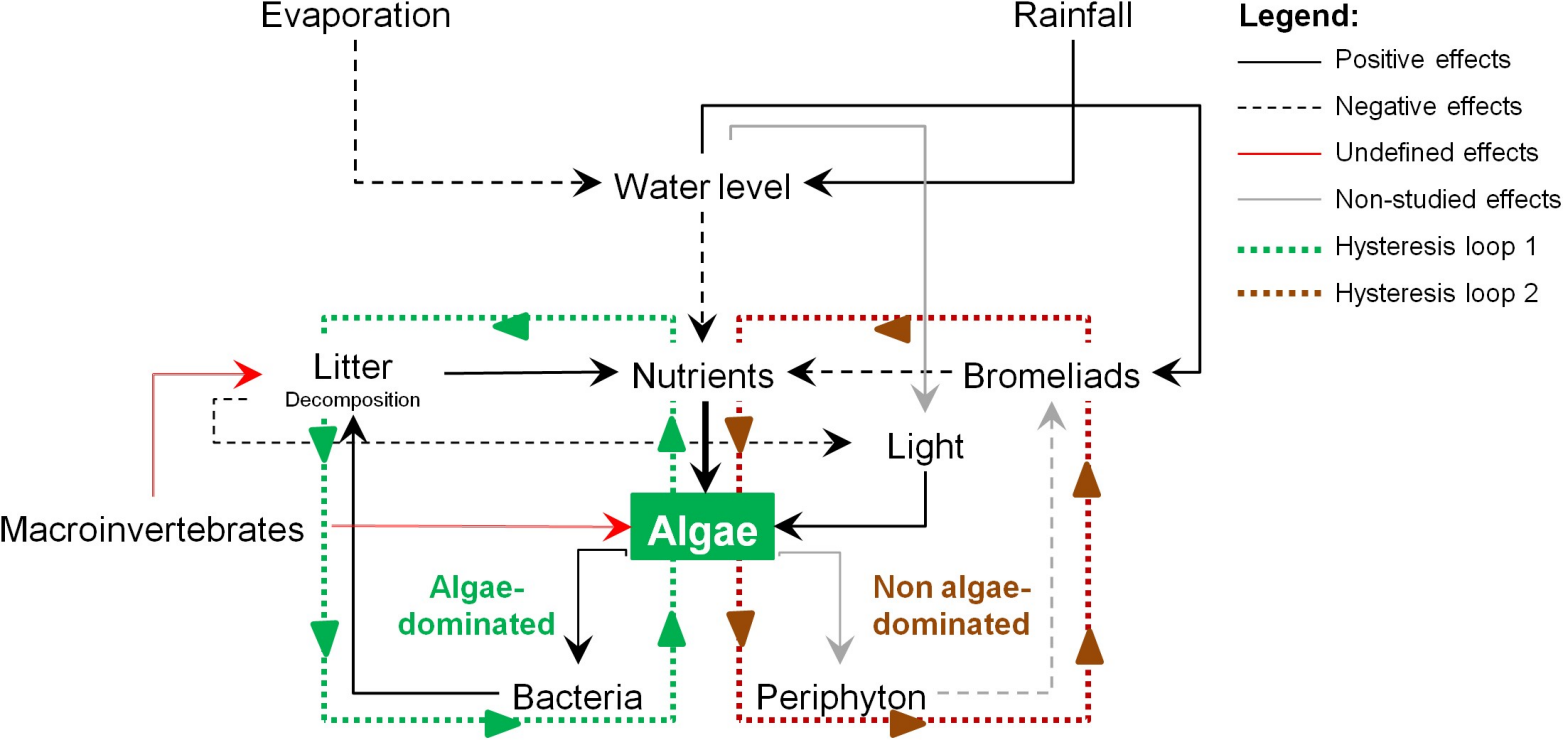
A conceptual model for alternative stable states in tank bromeliad ecosystems. Arrows represent direct effects of a variable on the other: continuous line (positive effects); dashed line (negative effects). Red lines represent the undefined effect of macroinvertebrates communities that will depend on several communities’ parameters, such as the number of trophic levels and community composition. Gray lines represent potential effects which were still not tested and should be incorporated in future studies. Lines with superimposed arrows demonstrate the hysteresis loops that determine the algae-dominated ecosystems (green loop) and non algae-dominated ecosystems (brown loop).

Our data showed that rainfall changes affected the occurrence of algae-dominated and non-algal conditions in tank bromeliads, which could be indirectly related to the water level in these ecosystems. Interestingly, our isotopic analysis revealed that high changes in rainfall amplitude (HA rainfall scenario) increased the content of ^15^N in the bromeliad leaves which suggests an enhanced ability in taking up nutrients from the water in these rainfall conditions. This result suggests that, in the Restinga of Jurubatiba, water can be a limiting factor for bromeliads to obtain nutrients. Since bromeliads are able to take up water and nutrients by their foliar trichomes and the availability of water increases the contact of trichomes with nutrients [31,49,50], an increment in the rainfall amplitude may thus increase the contact of trichomes with nutrients, increasing bromeliad nutrition. As consequence, we observed a negative correlation between the total amount of ^15^N in bromeliad leaves and the chlorophyll-*a* contents and algal biomass in the water in the tank. Previous studies have reported the interaction between bromeliads nutrition and the behavior of the aquatic ecosystem in their tanks [23,49,51]. Tank bromeliads shelter numerous microorganisms, algae and metazoan, and have to compete with them for the available nutrients present in the water [50–52]. Since the highest rainfall amplitude favors bromeliad nutrition, bromeliads seem to be the better competitors for nutrients than the algae in this condition. Thus, we suggest that changes in the water level are a crucial regulating factor of the algal biomass in the bromeliads tank, which can be indirectly related to bromeliad size [26] or with rainfall dynamics, as summarized in our proposed conceptual model (Fig 5).

Otherwise, it is important to highlight that each rainfall scenario is described by changes in frequency, intensity and clustering of rain events. For example, in *HA* rainfall scenario, tank-bromeliads have received the greatest rainfall input (100 mm) which implies that at in a short term, these ecosystems presented the wetter conditions and as consequence the most suitable habitat for colonization at this moment. As time goes by the absence of rainfall creates a drought condition which may trigger changes in the whole ecosystem and decrease the occurrence of algae-dominated ecosystems. Thus, *Ambient* rainfall scenario creates a more stable wet condition without great changes in the water level in these ecosystems. In this way, the observed effects of rainfall scenarios reveal the overall balance of all conditions described by them.

Litter diversity did not affect the algae-dominance in tank bromeliads nor minimized the effects of rainfall changes, as we first hypothesized. However, our qualitative approach over time demonstrated that litter diversity may have a positive effect on algae-dominated conditions in the *Ambient* rainfall scenario (see Supporting Information). In *Ambient* conditions, litter did not prevent light entering the system as the greater amount of water in this scenario raised the water level. On the other hand, in altered rainfall scenarios, bromeliad systems might have a limited amount of water and might be covered by the litter for an extended period. Additionally, litter decomposition could affect the turbidity of the water and consequently may also control light availability and the concentration of nutrients in the system (Fig 5). As a consequence, the high turbidity or the reduced light input promoted by litter in the tank will limit algal growth despite the availability of nutrients in the tank.

Based on the current experiment and on the mechanisms revealed by previous studies, we built a conceptual model to establish the potential applicability of the alternative stable states theory to tank bromeliads ecosystems. For that, we propose two self-reinforcing mechanisms, based on the current ecological knowledge for tank bromeliads, which could explain the maintenance of green and brown conditions in these systems. We suggest that bromeliads can play a comparable role as submersed macrophytes in shallow lake ecosystems [53] (Fig 5). Submerged macrophytes obtain their nutrients partially from the water, reducing the concentration of nutrients available for algae and microorganisms [23,49,51] and by doing so keeping the lake in a clear state. Bromeliads also take up nutrients from the water in their tank and thus also lower the available nutrients for algae and bacteria (Fig 5). Additionally, the presence of algae in the system may favor microbial activity [40]. This higher microbial activity increases the decomposition rate resulting in a higher release of nutrients which are then available for the algae. These feedbacks seem to reinforce the algae-dominated condition (hysteresis loop 1; Fig 5). Furthermore, periphyton may also have an essential role in regulating the ability of bromeliads taking up nutrients from the water. Periphyton grows on the surface of bromeliad leaves and takes up nutrients by itself, thus reducing the bromeliad ability to take up nutrients from their tank. However, the uptake of nutrients by bromeliads leaves may reduce the provision of labile algal dissolved organic carbon for periphyton. This would maintain the bromeliad leaf surface free of periphyton, allowing for continued removal of nutrients from the water column. These feedbacks may promote another self-reinforcing mechanism for the non algae-dominated condition (hysteresis loop 2; Fig 5). Finally, macroinvertebrates positively contribute to litter decomposition, having a positive effect on nutrient availability and likely in algae growth (Fig 5). However, filter feeding invertebrates may directly remove algae from the water column, but since these animals usually occur in low abundance in bromeliad ecosystems [16,48] it is very unlikely they largely affect algal biomass.

This is the first study that empirically demonstrates how changes in the rainfall and litter diversity may lead to shifts in algal conditions in tank bromeliad ecosystems. We propose some self-reinforcing feedback mechanisms (Fig 5) which make it plausible that those conditions might be alternative stable states. Previous studies attempted to apply the alternative stable state theory in natural microcosms, but they failed in proposing those self-reinforcing mechanisms [19,54]. We strongly suggest to further test the importance of the proposed feedback mechanisms in future studies. Finally, we conclude that changes in rainfall distribution might affect aquatic ecosystems by modifying its structure and the activity of the organisms therein which can trigger regime shifts or push environmental conditions closer to a tipping point.

## Supporting information

S1 Appendix Discussion about the temporal dynamic of the algae dominance in the tank bromeliads ecosystems, ^15^N enrichment of *Eugenia uniflora* leaves and tables with the statistic values of the analysis performed. (DOCX)

S1 Fig Effects of rainfall scenario and litter diversity on the frequency of algae dominance. We reported the frequency of algae-dominated ecosystems by the percentage of ecosystems that were considered algae-dominated, chlorophyll-*a* values higher than 80 μg L-1, in each litter diversity level for all rainfall scenarios. *Ambient*, Medium clustering (*MC*), Medium amplitude (*MA*), High clustering (*HC*) and High amplitude (*HA*) rainfall scenarios are fully described in the main text. * Non algae-dominated states found in the respective litter diversity and rainfall scenario. For statistical details see Table 1 in the main text.
(TIF)

S2 Fig Frequency distribution of chlorophyll-*a* (μg L^-1^) in the water of *Neoregelia cruenta* tanks in *Ambient* rainfall scenario. The lines depict the probability density described by the best fitting model (n = 35).
(TIF)
