# Supplementary material for: Rainfall changes affect the algae dominance in tank bromeliad ecosystems
Source: PLoS One. 2017 Apr 19;12(4):e0175436. doi: 10.1371/journal.pone.0175436 (PMC5396887; doi:10.1371/journal.pone.0175436)
Supplement: S1 Appendix — (DOCX) [file pone.0175436.s001.docx]

**Supporting Information**

**Temporal dynamics of algae-dominated tank bromeliads ecosystems**

We used a qualitative approach through visual observation of the water color to enable a temporal evaluation of the algal biomass in the tank bromeliads. Prior to the final quantitative sampling described in the main text, we performed three previous observations (90, 120 and 150 days), without directly to access chlorophyll-*a* concentration in the water. We determined the algae-dominated state by assuming those waters that were visually green. This is a very conservative criterion frequently used to classify shallow lakes as being turbid [1]. To ensure the reliability of our approach, we compared our observational data with the chlorophyll-*a* concentration values in the last sampling for the 175 tank bromeliads. All tank bromeliads that were visually assigned as algae-dominated had chlorophyll-*a* values higher than 80 µg L^-1^.

To verify the temporal consistency of this effect, we performed a Generalized Linear Mixed Model (GLMMs). We used a binomial distribution and designated the algae-dominated bromeliads by the value “1” and non algae-dominated state by the value “0”. We used each bromeliad replicate as a random factor because it allowed us to evaluate the consistency of the effects of each predictor irrespective to individual responses of the experimental microcosm. Time, rainfall scenario, litter diversity and the interaction between rainfall scenario and litter diversity were used as a predictor variable. To obtain the significance value for each predictor variable, we compared the models against a null model that included only the random factor. We carried out the GLMMs using the “*lmer*” package in R.

Rainfall changes (χ^2^=12.39, *P*=0.014) and its interaction with litter diversity (χ^2^=16.10, *P*=0.006) significantly affected the occurrence of non-algal states and consequently the algae-dominated state (Table C). Litter diversity positively affected the occurrence of an algae-dominated condition in the *Ambient* rainfall scenario and in *HA* rainfall scenario but presented different directions in the other altered rainfall scenarios (S1 Fig). Experimental time (χ^2^=0.01, *P*=0.900) and litter diversity (χ^2^=0.32, *P*=0.570) did not show any significant effects on the results (Table C).

Here, we demonstrated that the effects of litter diversity on the occurrence of algae-dominated ecosystems are dependent on rainfall scenario. Litter diversity positively affected the occurrence of green water bromeliads in *Ambient* rainfall scenarios, but this effect was lost in altered rainfall scenarios. These observations suggest that litter diversity effects should be depending on the maintenance of the aquatic condition that allows for aquatic organisms to respond to different litter mixtures [2]. Moreover, the effect of bromeliads in removing nutrients from the water is also dependent on the water condition. This highlights that the potential regulating effects of litter diversity on aquatic ecosystems can be dependent on the persistence of certain conditions [2], mainly processes depending on plankton dynamics, such as algal productivity. We also consider that litter may have a physical role in the light entrance in the tank of bromeliads which may limit the algae growth in them. *C. hilariana* litter is a waxy and heavy leaf that is deposited in the bottom of the tank, allowing light entrance but low nutrients loading. On the other hand, *E. uniflora* (C_3_) leaf litter is the most labile leaf litter, but it avoids light entrance in the tank as well as occurs in for *Cyperus sp.* (C_4_) because they can float on the water surface. As we increased litter diversity in a substitutive design, we allowed for a lot of nutrients provided by C_3_ litter, but there is still light incidence in the tank. This promotes light entrance, nutrients, time for colonization and the water condition necessary for algae establishment and growth. However, this effect was only observed as we increase the number of cases in our analysis by including all four sampling times (n=700), which suggest that this effect might be slight and extremely variable.

**^15^N enrichment of *Eugenia uniflora* leaves**

*Eugenia uniflora* plants were watered for 10 days with 300 mL of a labeled solution (5 g L^-1^ day^-1^) of ammonium sulfate (^15^NH_4_)_2_SO_4_ (10 atom % excess, from Cambridge Isotope Laboratories, Andover, MA, USA), as proposed by previous studies with tank bromeliads [3,4]. Labeled C_3_ leaves were dried at 60 ºC and distributed in the central portion of the bromeliad tanks at the beginning of the experiment, as previously described [5]. At the end of the experiment, we clipped two new bromeliad leaves from the innermost node of each experimental bromeliad for ^15^N analyses. Stable isotope ratios of ^15^N were determined in the Stable Isotope Facility Laboratory (UC Davis, CA, USA) using continuous flow isotope ratio mass spectrometer (20–20 mass spectrometer; PDZ Europa, Sandbach, England) after sample combustion to N_2_ at 1000 ºC by an on-line elemental analyser (PDZ Europa ANCA-GSL).

**Table A.** **Bayesian Information Criterion (BIC) values for chlorophyll-*a* in all rainfall scenarios.** BIC values for *Ambient*, Medium clustering (*MC*), Medium amplitude (*MA*), High clustering (*HC*) and High amplitude (*HA*) rainfall scenarios established using a different potential number of classes (k values). The lowest BIC value means the best number of classes that characterizes the response variable in each rainfall scenario (bold numbers). BIC values were obtained using the package “*flexmix*” in R Software.

| **Rainfall scenario** | **k values** | | |
| --- | --- | --- | --- |
|  | 1 | 2 | 3 |
| All | 420 | **-5335** | -5320 |
| Ambient | 95 | **-967** | -956 |
| MC | 92 | **12** | * |
| MA | 89 | **-1288** | -1277 |
| HC | 82 | **-1053** | * |
| HA | 78 | **-850** | -839 |

* Means that the number of classes proposed is not possible in the respective rainfall scenario.

**Table B. Generalized linear models for the effects of limnological variables on the occurrence of algae-dominated states.** Effects of water color, turbidity, dissolved nitrogen, phosphorus and organic carbon were evaluated. We used the binomial distribution to fit the models to our response variable. All models presented non-significant values (*P*>0.05).

| **Factor** | **d.f** | **χ^2^** | ***P*** |
| --- | --- | --- | --- |
| Water color | 1 | 0.82 | 0.365 |
| Turbidity | 1 | 0.44 | 0.506 |
| Nitrogen dissolved | 1 | 0.41 | 0.520 |
| Phosphorus dissolved | 1 | 0.00 | 0.976 |
| Carbon dissolved | 1 | 0.01 | 0.932 |

**Table C.** **General linear mixed models for the effects of time, rainfall scenario, litter diversity and the interaction between rainfall and litter diversity on the occurrence of algae-dominated ecosystems.** For this analysis, we used a categorical approach where algae-dominated ecosystems were characterized as full green water or those contained more than 80 µg L^-1^ (final sampling). Rainfall scenarios were *Ambient*, Medium clustering (*MC*), Medium amplitude (*MA*), High clustering (*HC*) and High amplitude (*HA*) and are fully described in the main text. In all models, bromeliad identity was used as a random factor. *P* values were obtained comparing models with the respective factor and a null model that include only the random factor. Significant values are presented in bold letters (*P*<0.05).

| **Factors** | **d.f** | **χ^2^** | ***P*** |
| --- | --- | --- | --- |
| Time | 1 | 0.05 | 0.815 |
| Rainfall scenario (RS) | 4 | **12.04** | **0.017** |
| Litter diversity (LD) | 1 | 0.25 | 0.617 |
| RS x LD | 5 | **15.53** | **0.008** |

**References**

1. Kosten S, Vernoij M, Van Nes EH, Sagrario M de LÁG, Clevers JGPW, Scheffer M. Bimodal transparency as an indicator for alternative states in South American lakes. Freshw Biol. 2012;57: 1191–1201. doi:10.1111/j.1365-2427.2012.02785.x

2. Pires APF, Marino NAC, Srivastava DS, Farjalla VF. Predicted rainfall changes disrupt trophic interactions in a tropical aquatic ecosystem. Ecology. 2016;97: 2750–2759. doi:10.1002/ecy.1501

3. Romero GQ, Srivastava DS. Food-web composition affects cross-ecosystem interactions and subsidies. J Anim Ecol. 2010;79: 1122–1131. doi:10.1111/j.1365-2656.2010.01716.x

4. Gonçalves AZ, Oliveira RS, Oliveira PS, Romero GQ. Species-Specific Effects of Ant Inhabitants on Bromeliad Nutrition. PLoS One. 2016;11: e0152113. doi:10.1371/journal.pone.0152113

5. Romero GQ, Mazzafera P, Vasconcellos-Neto J, Trivelin PCO. Bromeliad-living spiders improve host plant nutrition and growth. Ecology. 2006;87: 803–808. doi:10.1890/0012-9658(2006)87[803:BSIHPN]2.0.CO;2
