# Supplementary figures and images for: Rainfall changes affect the algae dominance in tank bromeliad ecosystems
Source: PLoS One. 2017 Apr 19;12(4):e0175436. doi: 10.1371/journal.pone.0175436 (PMC5396887; doi:10.1371/journal.pone.0175436)

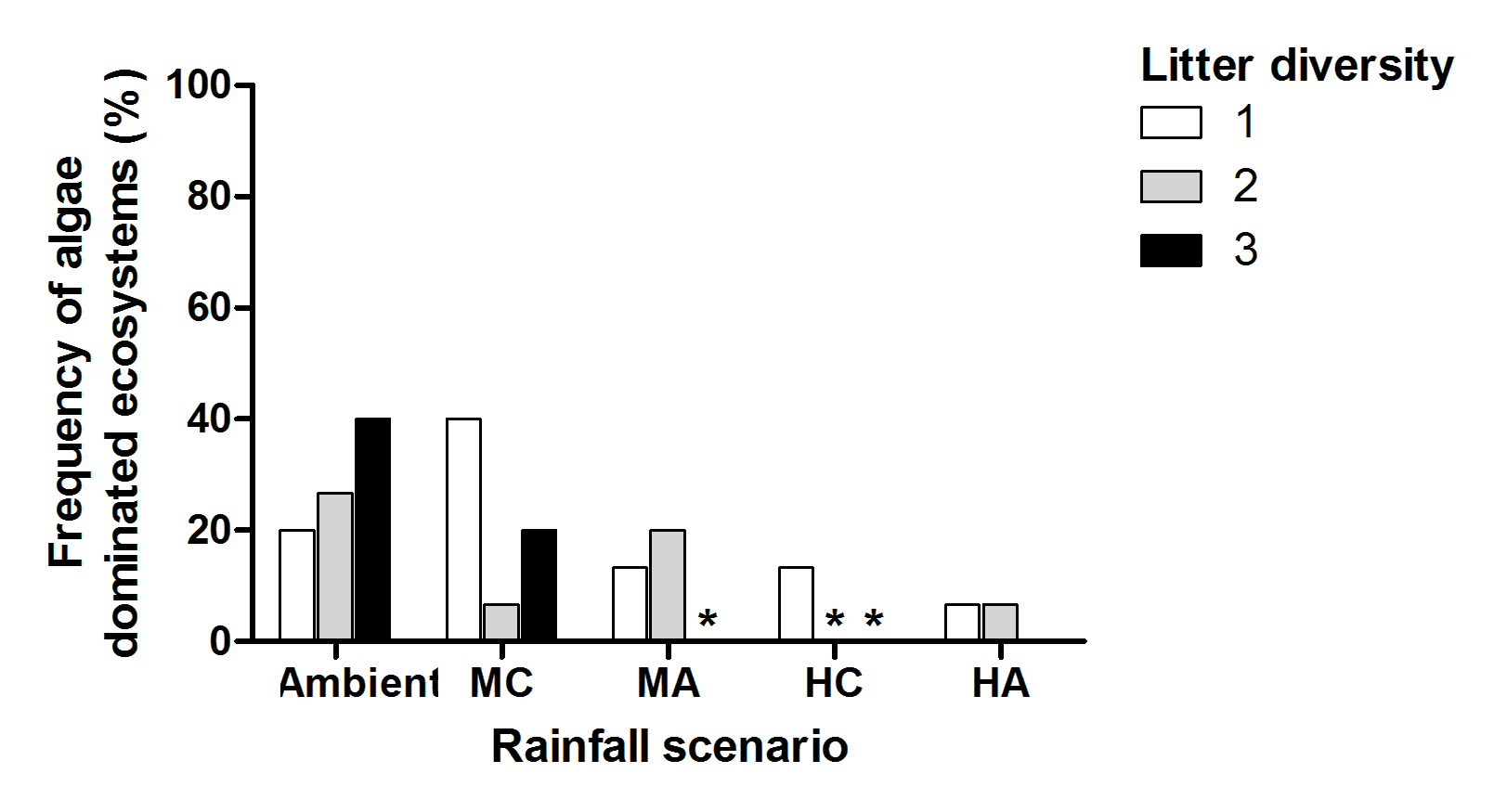

Supplement: S1 Fig — We reported the frequency of algae-dominated ecosystems by the percentage of ecosystems that were considered algae-dominated, chlorophyll-a values higher than 80 μg L-1, in each litter diversity level for all rainfall scenarios. Ambient, Medium clustering (MC), Medium amplitude (MA), High clustering (HC) and High amplitude (HA) rainfall scenarios are fully described in the main text. * Non algae-dominated states found in the respective litter diversity and rainfall scenario. For statistical details see Table 1 in the main text. (TIF) [file pone.0175436.s002.tif]

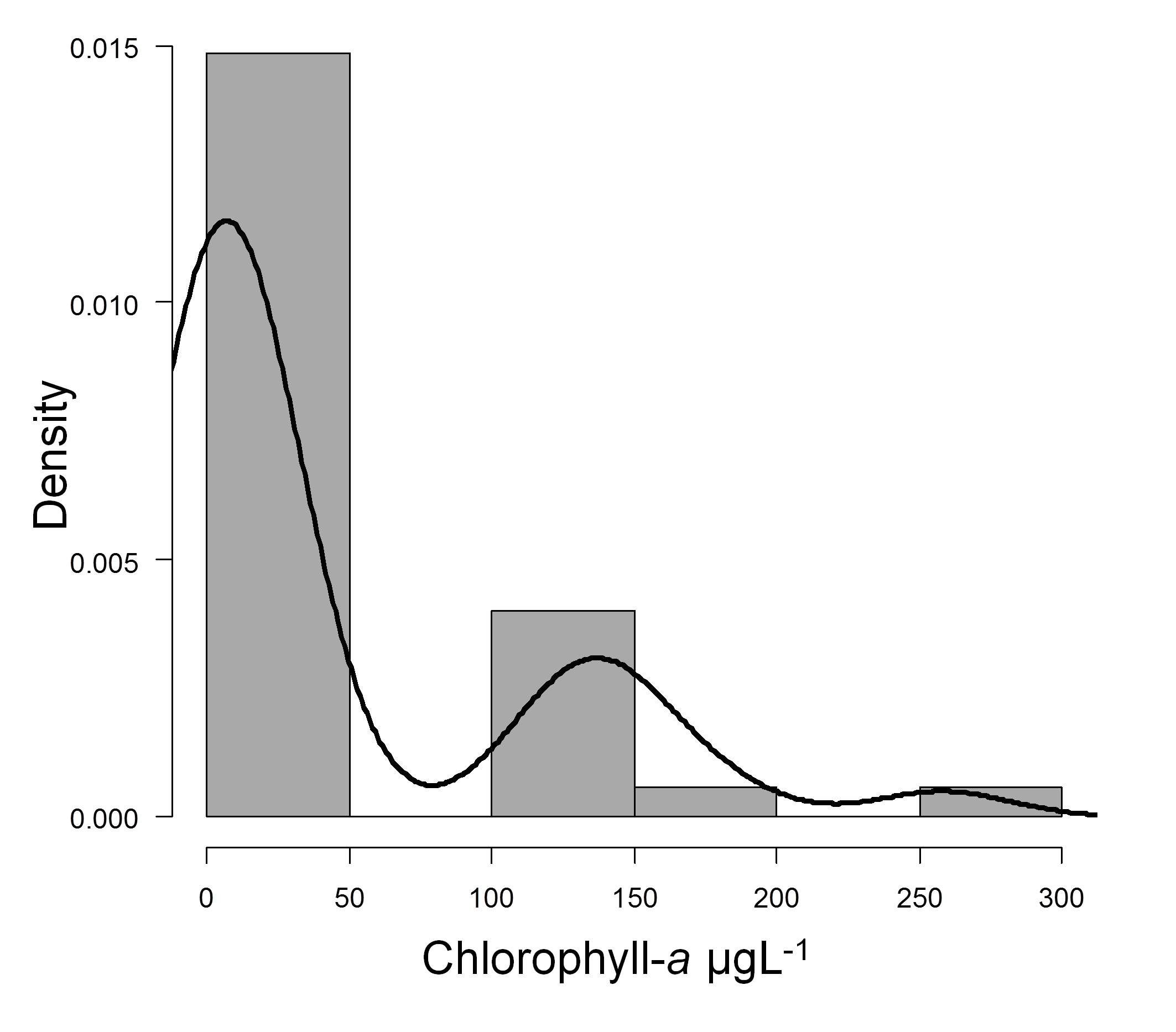

Supplement: S2 Fig — The lines depict the probability density described by the best fitting model (n = 35). (TIF) [file pone.0175436.s003.tif]
